# Paraplegia in a Patient With Tongue Cancer Caused by Metastatic Rib Tumor Extension Through an Intervertebral Foramen: A Case Report

**DOI:** 10.7759/cureus.77029

**Published:** 2025-01-06

**Authors:** Chihiro Kanno, Morio Yamazaki, Sadanoshin Yaginuma, Manabu Endo, Tetsuharu Kaneko

**Affiliations:** 1 Oral and Maxillofacial Surgery, Fukushima Medical University, Fukushima, JPN

**Keywords:** adolescent and young adult (aya), malignant spinal cord compression, paraplegia, rib metastasis, tongue cancer

## Abstract

Among patients being treated for cancer, almost all malignant spinal cord compression (MSCC) occurs in those with vertebral bone metastases. MSCC without vertebral bone metastasis is extremely rare; however, we report herein a case of rapid paraplegia caused by metastatic rib tumor progression through the intervertebral foramen in a patient with tongue cancer. A 37-year-old man was diagnosed with squamous cell carcinoma of the tongue (T2N0M0). The patient underwent a partial glossectomy, followed by neck dissection for cervical metastasis and anticancer drug therapy for lung and third rib metastases. The rib metastasis progressed, subsequently extending through the intervertebral foramen, in the absence of vertebral bone metastasis. At the time of detection, symptoms of MSCC were not observed. However, eight days after the diagnosis of tumor extension through the intervertebral foramen, slight paraplegia appeared, followed by complete paraplegia on day 10. Despite radiotherapy and glucocorticoid therapy, the paraplegia did not improve, and the patient died 90 days after the diagnosis of MSCC.

## Introduction

Malignant spinal cord compression (MSCC) is a serious oncological emergency requiring the implementation of rapid countermeasures. MSCC in head and neck cancer patients without vertebral bone metastasis is extremely rare [1]. Even when appropriate countermeasures are taken, however, MSCC often results in obvious impairment to quality of life (QOL), not only for patients with terminal cancer but also for their families and caregivers. We present herein a case of paraplegia that occurred over an extremely short period of time due to the progression of a metastatic rib tumor that extended through the intervertebral foramen in a patient with tongue cancer.

## Case presentation

**Figure 1:**
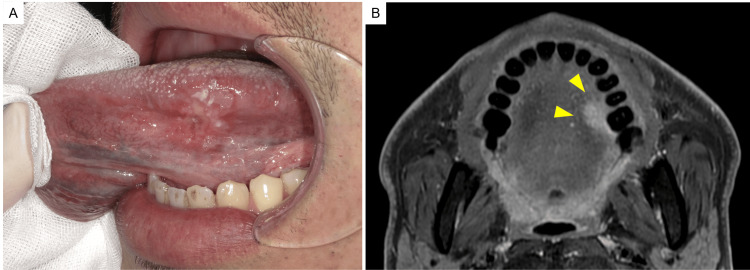
Primary disease at the first examination An unsmooth ulcer on the left side of the tongue on the first examination (A). Contrast-enhanced magnetic resonance imaging (MRI) showing an enhanced tumor in left side of tongue (arrow head) (B).

A 37-year-old man with no relevant medical history was referred to a local dental clinic in October 2018 with unhealed stomatitis and pain on the left side of his tongue. Mingled white and red ulcers were observed on his tongue. The patient was subsequently referred to our hospital. After evaluating the patient with contrast-enhanced computed tomography (CT), magnetic resonance imaging (MRI), and positron emission tomography/CT (PET/CT), the patient was diagnosed with T2N0M0 squamous cell carcinoma of the left tongue (Fig. 1A, 1B).

**Figure 2:**
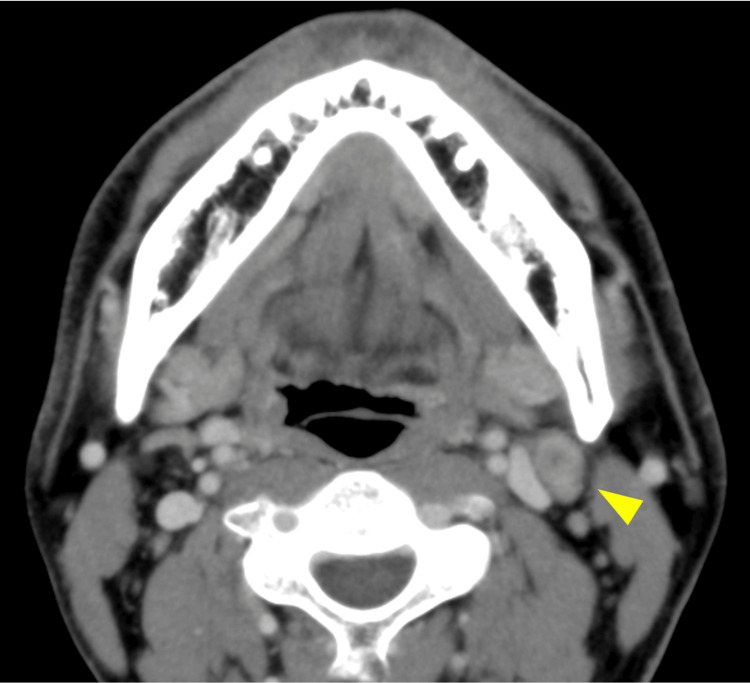
Imaging findings of late lymph node metastasis Contrast-enhanced computed tomography (CT) showing enlarged lymph node suspicious for central necrosis in level Ⅱ lesion (arrow head).

The partial glossectomy was performed in January 2019. The histopathological diagnosis of the resected margin was negative. Three months postoperatively, late lymph node metastasis was observed, after which a neck dissection was performed (Fig. 2).

**Figure 3:**
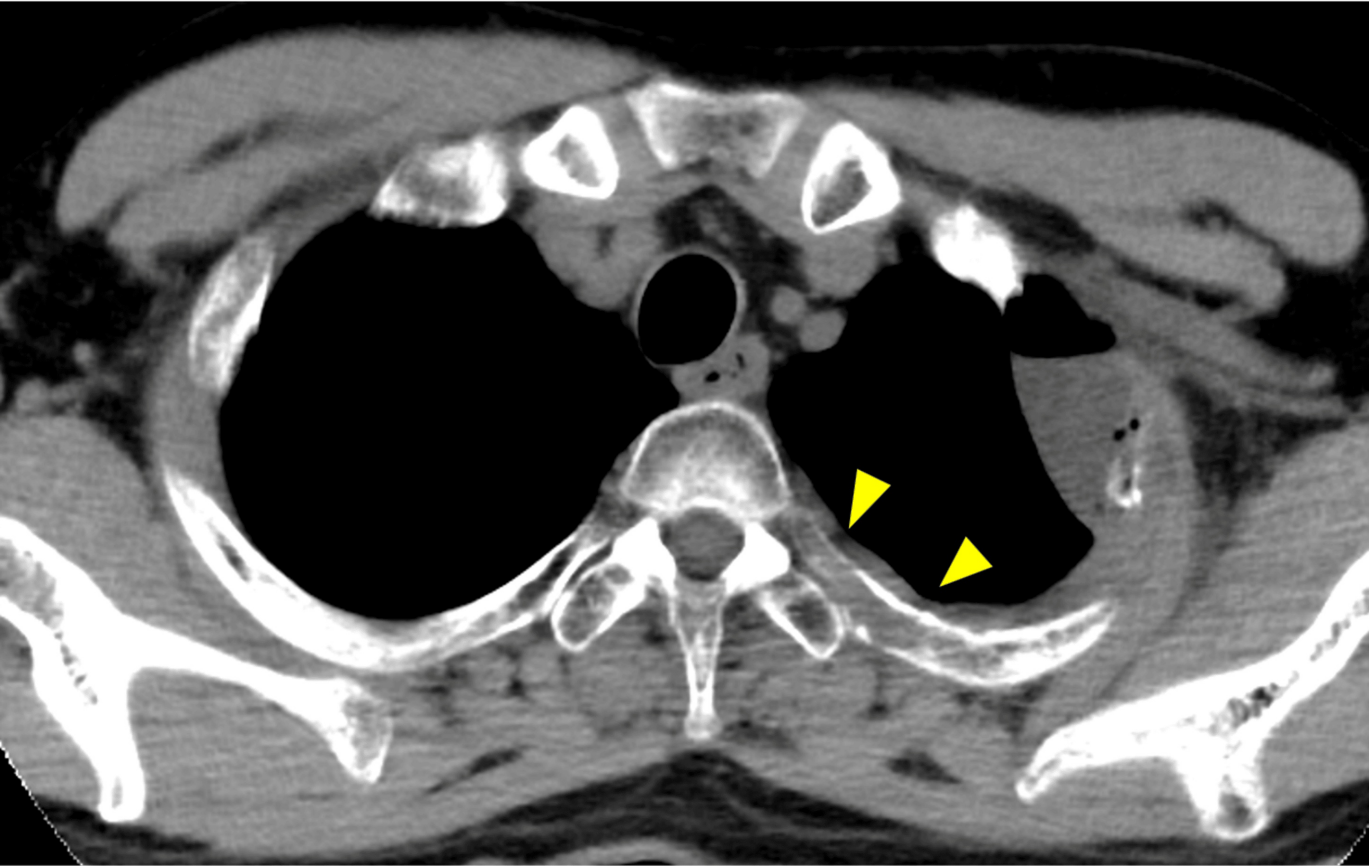
Image findings of rib bone metastasis CT showing the osteolytic lesion in the left third rib (arrow head) in April 2020.

Adjuvant concurrent chemoradiotherapy was administered to treat pathological extracapsular spread in the level II lymph nodes (60 Gy in 30 fractions, three courses of triweekly cisplatin 80 mg/m^2^). In October 2019, the locoregional status showed no recurrence; however, a metastatic tumor was observed in the left lung, for which nivolumab (biweekly, 240 mg/m^2^) was initiated. The treatment response was stable after six courses of nivolumab therapy, after which volumetric modulated arc therapy (VMAT) was performed (46 Gy in 23 fractions). In May 2020, the irradiated lung tumor was stable, although a new metastasis was observed on the posterior side of the left third rib with pain upon motion (Fig. 3).

**Figure 4:**
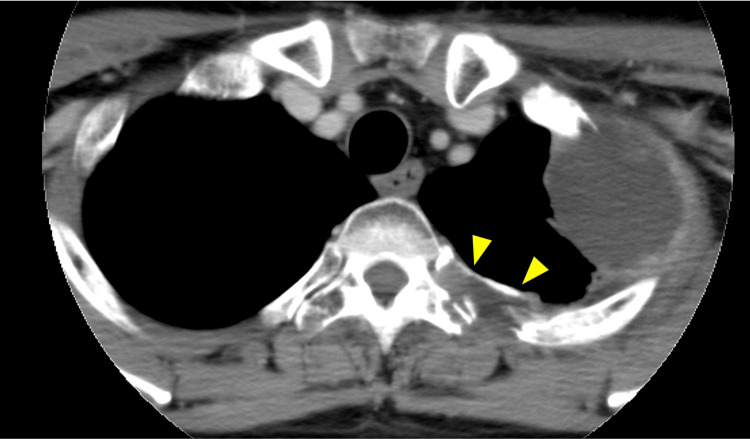
Image findings exacerbated distant metastasis CT showing exacerbation of the left third rib (arrow head) and lung metastasis with significant tumor enlargement in February 2021.

Weekly Cmab + PTX therapy (cetuximab, 400 mg/m^2^ in the first course and 250 mg/m^2^ for the second and subsequent courses; paclitaxel, 80 mg/m^_2_^ + subcutaneous denosumab 4 mg every four weeks) was initiated. The response was initially considered good; however, regrowth of the left third rib and lung tumor (Fig. 4) and new metastases were observed in the mediastinal lymph nodes in February 2021.

Subsequently, Pmab + FP therapy (pembrolizumab, 200 mg/body; cisplatin, 80 mg/m^2 ^+ 5 fluorouracil, 800 mg/m^2^) was initiated once the combined positive score from the resected cervical lymph node was 1-19.

**Figure 5:**
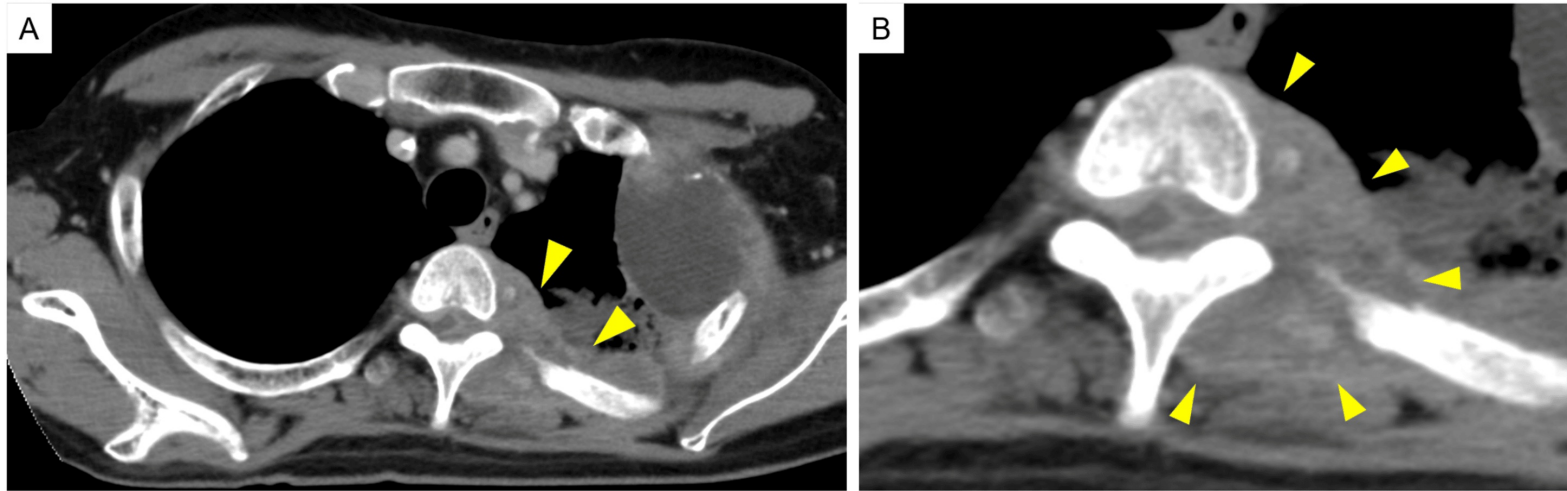
Image findings of metastatic tumors extended into the vertebral canal. Contrast-enhanced CT showing the metastatic third rib tumor extending into the vertebral canal through the intervertebral foramen between the second and third thoracic vertebrae (arrow head) (A). The magnification imaging of Fig. 5A shows the tumor mass compression of the spinal cord (arrow head) (B).

In June 2021, after six courses of Pmab + FP therapy, contrast-enhanced CT revealed further disease progression. In particular, the metastatic tumor on the third rib showed significant growth and had extended into the vertebral canal through the intervertebral foramen between the second and fourth thoracic vertebrae (Fig. 5A, 5B).

No osteolytic or destructive changes in the vertebral bones were observed. On that day (day 0), the orthopedic doctor observed no signs of spinal cord compression or exacerbation of back pain, and the radiologist prescribed palliative radiotherapy for two weeks. On the morning of day 8, sudden ambulatory weakness appeared, and the orthopedic physician diagnosed the patient with spinal cord compression. Emergency VMAT and steroid pulse therapy (methyl-prednisolone, 1,000 mg/day × two days) were administered. However, complete loss of feeling and motion of the lower legs and bladder and bowel dysfunction were observed on day 10. The patient was diagnosed with paraplegia due to spinal cord compression caused by the growth of the left third rib metastatic tumor. Nevertheless, a total of 49 Gy in 14 fractions of VMAT was administered, as was the oral dexamethasone (2 mg/day); however, the paraplegia did not resolve. The patient was discharged with home palliative care on day 22 and died on day 90.

## Discussion

In our patient, however, MSCC was caused by the extension of the tumor from the third rib into the spinal column. To the best of our knowledge, no previous reports of MSCC caused by this route have been published, making the present case extremely rare.

In OSCC (oral squamous cell carcinoma), distant metastasis is a rare event with an incidence of 1-4.1% [2,3]; the most common site is the lung (60%), then the bone (15%). Among the bone metastasis, the rib bone is less common. The risk factors are N states (presence or not) and T states (T3 and T4); especially, nodal metastasis is the most crucial factor of distant metastasis [2,3]. However, the present case was pathological T2 without metastasis in the first treatment, and late nodal metastasis with a pathological extra capsule spread was developed. Moreover, distant metastasis in the rib bone near the vertebral foramen, a rare site, resulted in a miserable course. In the imaging follow-up of OSCC, in order to detect the presence of distant metastasis, CT should cover not only primary and neck lesions but also the lung and liver.

In cases of MSCC caused by solid cancers, 98% are due to the destruction and collapse of metastatic tumors in the vertebral bone [1], which then compresses the spinal cord from the epidural space. In oral squamous cell carcinoma, distant metastasis is rare. In addition, MSCC can rarely occur due to intradural or intraspinal tumors; however, cases other than vertebral bone metastases or spinal tumors, as we have presented herein, are extremely rare, with only one relevant Japanese report [4]. In that case, thoracic lymph node metastasis from breast cancer extended into the vertebral canal through the intravertebral foramen, and moderate ambulatory weakness was observed. The patient immediately underwent decompression and spinal fusion surgery followed by chemotherapy. She was able to walk a short distance, with a survival of over two years.

The efficacy of radiotherapy for the treatment of MSCC has been previously reported. The percentage of patients without paralysis due to MSCC who are ambulatory after irradiation is 92-100%, compared to 38-59% for patients with incomplete paraplegia, and only 10-17% of patients with complete paraplegia, suggesting that the early introduction of radiation therapy is effective [5-12]. However, these percentages include all fixed cancer metastases to the vertebral bones, and it is possible, therefore, that the outcomes for tumors such as squamous cell carcinoma of the head and neck, which have relatively rapid progression, may be inferior to those reported. In the present case, the patient did not show any symptoms of spinal cord compression, although extension into the vertebral canal was detected on CT, resulting in the delayed initiation of radiotherapy. Although this is a rare form of extension, we believe that radiotherapy should be initiated early in similar cases, even in the absence of symptoms of spinal cord compression.

Furthermore, many reports on the use of high-dose glucocorticoids do not recommend their aggressive use because of their low effectiveness in improving treatment prognosis and concerns about the possibility of adverse events, such as gastrointestinal ulcers and steroid psychiatric disorders [8,13-15]. In the present case, we used glucocorticoids with the aim of maintaining activities of daily living, even if only slightly, as the patient was young; although no efficacy was observed, evidenced by minimal reports of efficacy [16]. Although no adverse events were observed, glucocorticoids should be administered with extreme caution.

In the case presented herein, the first imaging evaluation was performed 16 weeks after the initiation of Pmab + FP therapy, for which six courses comprise the standard regimen [17], since there was no worsening of symptoms or intolerance to adverse events. However, clinical trials of this treatment regimen often include imaging evaluations every 8 weeks [17,18]. Moreover, as there were no options for further treatment, the patient completed all six courses without imaging evaluation. It is possible, however, that had an imaging evaluation been performed in the middle of the six courses, the radiotherapy could have been initiated earlier, which may have contributed to maintaining the patient’s end-of-life QOL. Therefore, frequent imaging evaluations during drug therapy for recurrent or distant metastatic head and neck cancer are necessary not only to evaluate the efficacy of the current therapy or the need to move the next therapy but also to assess tumor spread to dangerous areas in order to respond to oncological emergencies as early as possible.

## Conclusions

We described herein a case of paraplegia due to compression of the thoracic spinal cord by an enlarged rib metastasis. Palapregia might occur without vertebral bone destruction. When tumor extension into the perispinal cord is observed, it is necessary to intervene as soon as possible, even if vertebral bone metastasis is not observed.
